# Deciphering urban consumer requirements for rice quality gives insights for driving the future acceptability of local rice in Africa: Case study in the city of Saint‐Louis in senegal

**DOI:** 10.1002/fsn3.2136

**Published:** 2021-01-21

**Authors:** Ibrahima Mané, Joseph Bassama, Moussa Ndong, Christian Mestres, Papa Madiallacké Diedhiou, Geneviève Fliedel

**Affiliations:** ^1^ Route de Ngallèle Université Gaston Berger de Saint‐Louis Saint‐Louis Sénégal; ^2^ CIRAD UMR QUALISUD Montpellier France; ^3^ Qualisud CIRAD Montpellier SupAgro Univ d'Avignon Univ de La Réunion Montpellier France

**Keywords:** consumer acceptability, focus group discussion, local versus imported rice, quality, Saint‐Louis, Senegal

## Abstract

Rice is the staple cereal in Senegal. Despite the many policies implemented over the last decade, Senegalese consumers still prefer imported over local rice. To understand this preference, this study compares consumer acceptability of three local rice samples versus two imported rice samples. Two focus groups and a consumer test with 120 women were carried out in the city of Saint‐Louis in Senegal. The results concerning consumption habits showed that about 85% of the surveyed women consume rice at least once a day (at lunch). The hedonic test showed that consumers appreciated all five rice samples, but the most liked samples were obtained from industrial processing of either local or imported whole and fragrant rice. The least liked sample was a local semi‐industrial rice, including 50% broken grains. The results of the just‐about‐right (JAR) test and check‐all‐that‐apply (CATA) test showed that the sensory descriptors such as white color, well‐cooked, and homogeneous grain size had an influence on the consumers’ choice of rice samples. However, the most important selection criteria were the homogeneous size of the rice grains, the absence of impurities, both of which are directly linked to the milling conditions, and fragrance, which is related to the variety. The origin of the rice samples did not influence the consumers’ choice. This study showed that local rice can compete with imported rice if processing is improved in some small‐scale rice mills.

## Graphical abstract

The article compares the consumer acceptability of local rice versus imported rice. The sensory descriptors influencing the choice of the consumers were identified. Local rice can compete with imported rice.

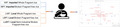

## 1 INTRODUCTION

Rice is one of the staple cereals in the Senegalese diet. At an estimated 50kg per capita per year (USDA, 2019), rice represents 34% of national cereal consumption (Maïga et al., 2020). It is used to prepare the main popular dishes, such as the famous “rice and fish” (*ceebu jen*), and is mainly consumed for lunch and/or dinner. With a growing population and increasing demand, Senegal currently has to resort to massive rice imports (Soullier et al., 2020), meaning the economy depends to a great extent on the international market (Colen et al., 2013). In this context, the Senegalese government has encouraged efforts to develop the sector and to move toward rice self‐sufficiency (Demont & Rizzotto, 2012). Since the 1980s, the government has invested in fertilizers, finance, and agricultural infrastructure via the National Society for the Development and Exploitation of the Senegal River Delta (SAED). Many policies were set up to improve rice production and consumption. These actions increased local rice production from 300 000 tons of paddy in 2009 to 1 007 277 tons in 2018 (ANSD, 2018). The majority of national rice production is harvested in the irrigated areas of the Senegal River valley. However, despite these efforts, the quality of the local rice still does not meet consumer requirements. The policies did not account for the impact of quality on the competitiveness of local rice compared with imported rice in urban markets (Lançon et al.., 2004). As a result, local rice is still unable to compete with imported rice (Demont & Rizzotto, 2012).

To understand Senegalese consumers’ demand, several studies based on the experimental auction method and surveys have been carried out to identify the criteria that influence their choice. Rutsaert et al. (2013) showed that the Senegalese are big consumers of imported fragrant 100% broken rice. These results were particularly true in urban areas (Colen et al., 2013; Rutsaert et al., 2013). Fall et al. (2007) also showed that a significant proportion of urban consumers were not even aware that valley rice exists. This situation could be mainly due to the limited availability of rice, the lack of awareness, and ineffective promotion of local rice. In urban centers such as Dakar, the population has a strong preference for imported broken rice that is cleaner (absence of impurities, black grains, dust, etc.) and more homogeneous (Fall et al., 2007), compared to local rice which consumers consider to be of inferior quality (Demont & Rizzotto, 2012). Most local paddy rice is milled into white rice in small local mills that are not equipped to produce high‐quality milled rice in terms of homogeneity and purity. Local rice contains a large proportion of impurities such as stones and dirt (Demont et al., 2013), quality defects due to technological limitations. Cooking properties are also important determinants of rice quality for consumers. Lançon and Méndez del Villar (2012) attest to the fact that, in West Africa, swelling capacity and cooking properties are the main criteria that determine rice consumers’ preferences.

The studies cited above were mainly based on socioeconomic analyses. Sensory criteria were hardly or not at all taken into consideration to explain consumer acceptance. Identifying these criteria through sensory mapping could complete socioeconomic studies and provide a more precise understanding of consumer demand by linking consumer overall liking to the physical–chemical and/or the sensory characteristics of the rice (Thomas, 2016). In Ghana, a study on urban consumer preferences and sensory evaluation of local versus imported rice showed that the sensory descriptors such as uniform appearance, attractive odor, rice odor, creamy flavor, and sweet taste were associated with imported rice, and the sensory descriptors such as hard texture, grainy texture, sour taste, brown color, and brown specks were associated with local rice (K. I. Tomlins et al., 2005). In Nigeria, where imported rice is the most popular, consumer acceptance was mainly related to rice sensory and physical properties of the grain including cleanliness, homogeneity, moisture content, and taste (Lançon et al., 2004). Conversely, results in Guinea and Mali showed that local rice was preferred for its taste and freshness (Rutsaert et al., 2013). A number of articles have been published on consumer acceptability of local products using hedonic test (Adinsi et al., 2015; Akissoé et al., 2015; Bechoff et al., 2014; Neville et al., 2017; K. Tomlins et al., 2007; Touwang et al., 2015). In the last decades, check‐all‐that‐apply (CATA) question has been widely used to provide valid sensory characterization of many different foods. It is a rapid sensory profiling tool that can be applied by consumers to describe, discriminate, and identify the drivers of liking (Aguiar et al., 2020; Bruzzone et al., 2015; Jaeger, Hunter, et al., 2015; Mello et al., 2019; Oliver et al., 2018; Pramudya & Seo, 2018; Tarancón et al., 2020; Verwaeren et al., 2019; Waehrens et al., 2018) . To date, no study has been conducted in Senegal to identify the links between the type of rice and their sensory characteristics.

The aim of this study was thus to identify the criteria that guide the choice of type of rice by consumers in an urban area (the city of Saint‐Louis, in the Northwest region of Senegal) by comparing the acceptability of local versus imported rice, of whole versus broken rice and of fragrant versus nonfragrant rice. The sensory descriptors were identified through focus group discussions followed by a consumer test, using hedonic, JAR, and CATA tools. Deciphering the demand for quality by Senegalese consumers provides valuable information on how to improve the quality and sales of local rice.

## 2 MATERIALS AND METHODS

### 2.1 Rice samples

Five rice samples (local and imported) were used in this study (Table 1). The local industrial rice was purchased both from retailers in the Saint‐Louis region and at the production sites in the case of the local semi‐industrial rice in the Saint‐Louis region. Imported rice was purchased from retailers in Dakar, the capital, because of the greater choice available.

**TABLE 1:** List of rice samples

Rice samples*	Origin	Type of Rice	Milling process	Fragrant
LsiBFf	Local Rice/Senegal River Valley	50% broken	Semi‐industrial	No
LWF	Local Rice/Senegal River Valley	Whole grains	Industrial	Yes
LBFf	Local Rice/Senegal River Valley	100% broken	Industrial	No
IBFf	Imported Rice/India	100% broken	Industrial	No
IWF	Imported Rice/Thailand	Whole grains	Industrial	Yes

* IWF: imported whole fragrant rice; LWF: local whole fragrant rice; LBFf: local broken fragrant‐free rice; IBFf: imported broken fragrant‐free rice; LsiBFf: local semi‐industrial broken fragrant‐free rice.

### 2.2 Cooking of the rice samples

All the rice samples were cooked in water under the same conditions and at the same time, using electric rice cookers (Tristar, Netherlands; one per rice sample) to facilitate rice preparation for consumer tests, and to avoid differences in the cooking of the rice. The consumption model chosen was white rice cooked in water, because it is one of the most widespread ways of cooking rice in Senegal. The cooking conditions were determined after several preliminary tests: 300g of rice was washed in 500 ml of tap water at room temperature for 5 min, drained in a colander, and placed in a rice cooker and 570 ml of water (water/rice ratio of 1.9), and 2g of salt were added. Each rice sample was cooked in a rice cooker until the water was completely absorbed. The rice was then transferred to an isothermal container until the consumer test.

### 2.3 Focus group discussions

Two focus group discussions (FGD) were organized in the Sanar district (Eastern part of Saint‐Louis city), the first one with nine female students (20 to 27 years old) and the second one with eight women (40 to 60 years old). In Senegal, in most households, the women buy, cook, and consume rice; they thus know the product very well; and they were well placed to give relevant information on rice and a list of more appropriate descriptors to describe its sensory properties.

In preparation for the focus group discussions, the lighting and temperature in the room were adjusted to be sure the conditions were favorable for the participants to describe the rice samples. Two food scientists, a PhD student in food science, and four trainees (BSc) facilitated the focus group discussion. The dialogue between the facilitators and the participants was conducted in French or in Wolof, the national language of Senegal. First, questions concerning rice consumption habits (choice of the type of rice, frequency of consumption, who bought the rice, how much they bought and where) were asked. Then, participants were asked about the usual ways rice is cooked in Senegal, what precautions are taken, and in particular, the steps that require the most attention when cooking rice. Finally, five samples of uncooked and cooked rice were presented to the women who were asked to describe the rice accurately and precisely to collect as many terms as possible to be used in the consumer test questionnaire. A total of 24 descriptors (Table 2) were cited by the participants.

**TABLE 2:** List of rice descriptors cited by women during FGD

Sensory descriptors	Description
Broken grains	Small grain size. Rice grains broken during milling
Whole grains	Grains that are completely intact after milling, as opposed to broken grains
Pasty texture	Contains a lot of water
Heterogeneous size	Mixture of grains of different sizes
Medium grains	Intermediate size grains
Easy to digest	Easy to digest after consumption
Hard texture	Difficult to eat (too firm texture)
Good taste	Characteristic taste of good rice
Fine grains	Long slender grains
Too many impurities	Bad postharvest and milling conditions
Too small grains	Presence of too many small rice grains
Soft texture	Soft, too easy to eat
Fragrant	Aroma of fragrant rice
White color	Characteristic color of white rice
Overcooked	Rice cooked for too long
Sticky texture	Cooked rice grains stick together or to the fingers
Well‐cooked	Good cooking
Old aftertaste	Taste characteristic of old rice or bad storage conditions
Scattered	Rice grains dispersed after cooking
Typical rice odor	Characteristic smell of good rice
Clean	No impurities
Good for ceeb	Suitable for the preparation of the popular Senegalese dish: rice with fish
Beautiful	Nice appearance
Dirty white	Characteristic color of old rice or due to the presence of dust

### 2.4 Consumer acceptability test

Consumer testing was conducted with 120 consumers, only women, in three districts of Saint‐Louis city: Sanar (*n* = 40), Balacoss (*n* = 40), and Guet‐Ndar (*n* = 40). The consumers, mainly from Senegal, who live in Saint‐Louis, were invited to participate in the consumer test. They were asked to answer a questionnaire, give personal information (gender, region/country of origin, nationality, country of residence, ethnic group, age, education, occupation, marital status, wealth status) and rice consumption habits (frequency, mode of consumption, when, where and with what other ingredients). Then, they were invited to taste the five selected rice samples in random order, one after the other, using three successive tests. First, a JAR “just‐about‐right” test carried out on three descriptors identified as important by the FGD participants (color, grain size, and degree of cooking) and scored by consumers using a three‐point JAR scale: "Too little," "Just About Right," and "Too much" (Li et al., 2014). Second, a hedonic test to assess the overall liking of each rice using a nine‐point hedonic scale (1 "extremely dislike" to 9 "extremely like") (Peryam & Pilgrim, 1957). Third, a “check‐all‐that‐apply” (CATA) table with 24 sensory descriptors (positive and negative) cited during the FGD, to better describe each rice sample by selecting exactly the right descriptors in the table (Ares et al., 2010; Ares & Jaeger, 2013; Espitia‐López et al., 2019; Jaeger, Beresford, et al., 2015). Finally, each consumer was asked to give her opinion and preferences on rice samples, asking her which rice is most likely the one she usually consumes, which rice she dislikes the most, and which she likes the most and why. All the interviews were conducted in French or in Wolof, with the questionnaire including personal information and the three tests, written in French. Consumers were assisted by the investigators when necessary. The test did not last more than 30 min.

## 3 ETHICAL ASSESSMENT AND CONSENT

Focus group discussions and the consumer test were approved by the Ethics Committee of the Cheikh Anta Diop University of Dakar. A consent form was signed by each participant. The rice samples were prepared according to good hygienic and manufacturing practices. Participants were informed about the study, that their participation was voluntary, and that they could stop the interview at any time if they wished.

### 3.1 Data analysis

An analysis of variance (ANOVA) was performed on the hedonic overall liking scores of each rice sample. Tukey's test was used to make multiple pairwise comparisons with a 95% confidence interval (P‐value < 0.05). A multiple factor analysis (MFA) and a principal component analysis (PCA, covariance) were conducted on the frequency of citations of the CATA descriptors used by the consumers to describe the five rice samples. Q Cochran's test was used to identify significant differences in the frequency of citations of each descriptor. All statistical analyses were performed with XLSTAT version 2019 (Addinsoft Inc, New York, USA).

## 4 RESULTS AND DISCUSSION

### 4.1 Information collected in the focus group discussions

During the focus group discussions, 75% of women mentioned that rice is consumed at least once a day by the Senegalese, and the remainder said it can even be eaten twice a day. In their opinion, rice is consumed so frequently because it is the staple food (*ceebu jen*) in Senegal, it is cheap and easy to prepare. In most cases, buying rice is the mother's responsibility, but the head of the family decides what type of rice to buy. Where the rice is bought depends where people live. Those who live near rice production areas often buy the white rice directly from industrial rice milling companies, from small‐scale rice mills (semi‐industrial rice), or from wholesalers (at the market). People who live in cities away from the production areas prefer to buy rice from shopkeepers or wholesalers (at the market). In addition, 70% of the women buy 50 kg bags at the end of the month, while others prefer to buy 2 to 3 kg per day, to avoid having to store the rice. The majority of consumers are loyal to a brand of rice. In the second FGD, 66% of women aged 40–60 years, who live in Saint‐Louis, said they always buy the same brand of local rice, whereas in the first FGD, 77% of the young female students at the University of Saint‐Louis who come from different regions in Senegal mentioned that they mainly bought imported rice. Indeed, they said they did not know how to cook local rice that is too dry and needs prior soaking or more water to cook. However, the older women are used to and prefer their local rice, they know where it comes from, and they cook it longer with or without prior soaking, they also know how much water to use.

Regarding uncooked rice, 77% of the women prefer the industrial local whole fragrant rice for its size, its fragrant smell, good for *Yassa* (white rice to accompany *Yassa,* a dish of chicken with a lemon sauce), the absence of impurities, nonsticky. They do not like local rice processed semi‐industrially because it is not white, contains too many impurities, smells of dust, and is difficult to cook. Regarding cooked rice, 44% of the younger women prefer the imported broken rice because it is more presentable, tender, and has a small taste of natural rice. Thirty‐three percent chose the industrial whole fragrant rice from the valley rather than imported broken rice because they found the imported broken rice too sticky. Finally, 83% of the women aged 40 to 60 who live in St‐Louis prefer the industrial local whole fragrant rice because they said the imported broken rice was overcooked and the shape of the rice was no longer visible.

### 4.2 Rice consumption habits in Saint‐Louis region

During consumer testing, 120 women were interviewed in three different locations in Saint‐Louis city. Their socioeconomic profile and rice consumption habits are presented in Table 3. The women interviewed were between 18 and 60 years old, the majority (40%) between 30 and 49 years old. They had different levels of education depending on the district. In Sanar district, most of the women were interviewed at Gaston Berger University, explaining why 50% of them were quite young and had a higher level of education (university graduates). The results on consumption habits confirmed those obtained in the FGD. On average, 85% of respondents eat rice at least once a day (at lunch), with a stronger preference for fragrant‐free local broken rice (73%). Rice cultivation is well developed in the Senegal River valley and in particular in the Saint‐Louis region, thanks to the investments made by the government to improve hydro‐agricultural infrastructure, water management, technical supervision, research, and access to credit. According to Fall et al. (2007), the consumption of a type of rice depends on several factors, including the origin, the household income, and the type of rice available in the area over the year. These results are in line with those obtained by Colen et al. (2013) and Rutsaert et al. (2013) who showed that in river valley semi‐urban centers such as Saint‐Louis and Podor, consumers largely prefer local rice over imported rice. This accounts for 78% to 100% of the population's consumption. These results are also in agreement with those of Tomlins et al. (2005) who pointed out that consumers in Ghana tended to like the local rice samples that came from their own region.

**TABLE 3:** Socioeconomic profile and rice consumption habits of women consumers interviewed in Saint‐Louis city

	Total		Saint‐Louis districts (%)		
	%	Number	Sanar	Balacoss	Guet‐Ndar
Women interviewed (*N*)		120	40	40	40
Age					
18–29	26	31	48	18	13
30–49	40	48	25	48	48
50–69	34	41	28	35	40
Marital status					
Single	28	34	55	25	5
Married	66	79	38	70	90
Widow	6	7	8	5	5
Level of education					
No education	25	30	10	10	55
Primary education	35	42	30	48	28
Secondary education	13	15	8	20	10
High school diploma	12	14	8	23	5
University degree	16	19	45	0	3
Frequency of rice consumption					
Every day: twice a day	8	9	10	5	8
Every day: once a day	83	99	80	88	80
Several times a week	8	9	8	5	10
Once a week	3	3	3	3	3
Type of rice consumed					
Broken, fragrant‐free local	73	87	68	80	70
Broken, fragrant‐free imported	10	12	18	3	10
Broken, fragrant local	6	7	3	5	10
Broken, fragrant imported	11	13	25	3	5
Whole, fragrant‐free local	72	86	60	83	73
Whole, fragrant‐free imported	3	3	5	0	3
Whole, fragrant local	7	8	5	5	10
Whole, fragrant imported	2	2	3	0	3

### 4.3 Overall assessment of rice samples

An analysis of variance (ANOVA) was performed on the hedonic overall liking scores of each rice sample. Tukey's test revealed significant differences between the rice samples (P‐value < 0.05) (Table 4 **)**. All the rice samples were acceptable (scores above 5, neither like nor dislike), but four significant groups were identified. The most liked samples (Group A) were imported and local industrial whole fragrant rice (IWF and LWF), followed by (groups B and C), the imported and local broken fragrant‐free rice (IBFf and LBFf), and the least liked was a local semi‐industrial broken fragrant‐free rice (LsiBFf). These results show that the acceptability of the rice was not linked to the origin of the rice (local or imported). In fact, if rice samples of the same type (whole or broken grains) are processed similarly, they are appreciated in the same way by Senegalese consumers. On the other hand, if the rice is milled locally by small‐scale rice millers at a semi‐industrial scale, it is a little less appreciated. These results are in agreement with the statements made by the women during the two FGDs when they tested the five cooked rice samples. They said that the four industrial rice samples were their favorite, because the two whole fragrant types of rice are usually used to accompany a dish with a sauce (e.g., *yassa*), while the broken fragrant‐free rice is highly valued for a popular fish rice dish (*ceebu jen*). They ranked the local semi‐industrial 50% broken rice lower, because of its different sized grains, its dirty white color, and the presence of impurities. All these results confirm those obtained in recent experimental studies showing that local African rice can compete with imported rice if the quality meets consumer demand (Demont et al., 2013).

**TABLE 4:** Tukey test results on the comparison between products

Rice samples*	Mean overall liking	Standard error	Groups		
IWF	7.766	0.15	A		
LWF	7.552	0.15	A		
LBFf	6.766	0.15		B	
IBFf	6.112	0.15		B	C
LsiBFf	5.991	0.15			C

* IWF: imported whole fragrant rice; LWF: local whole fragrant rice; LBFf: local broken fragrant‐free rice; IBFf: imported broken fragrant‐free rice; LsiBFf: local semi‐industrial broken fragrant‐free rice.

### 4.4 Just‐About‐Right (JAR) test

**FIGURE 1:**
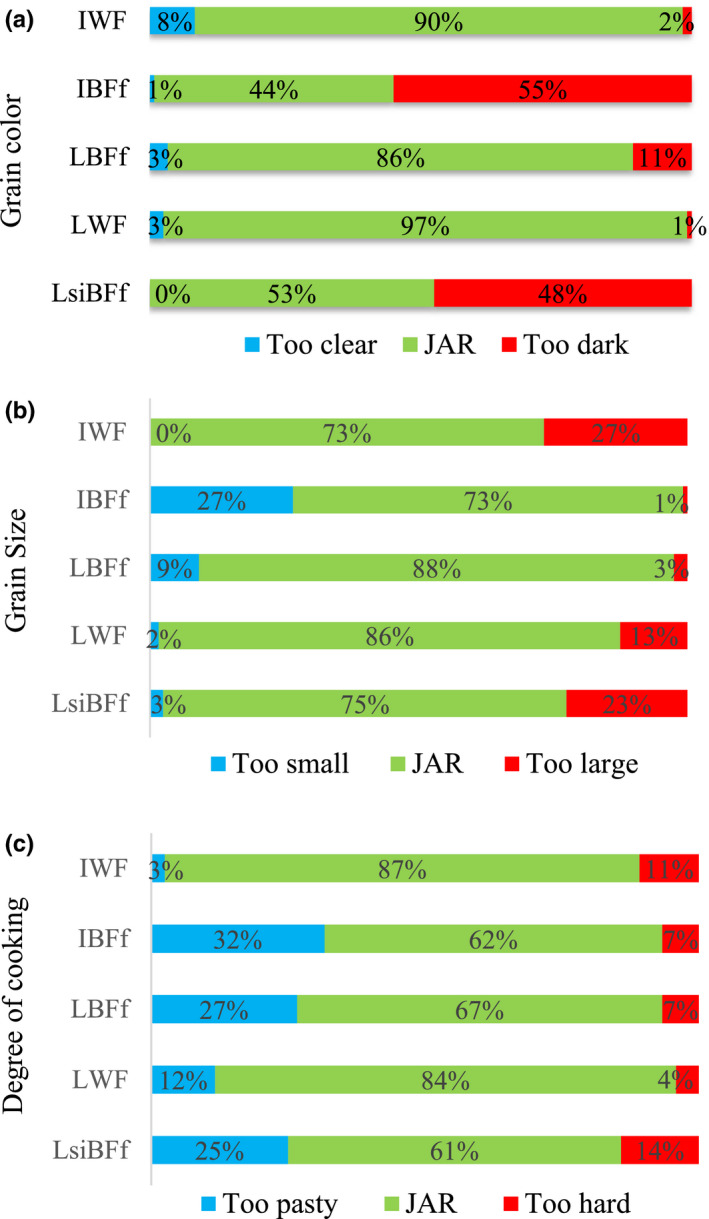
Just‐about‐right (JAR) test on three sensory descriptors (IWF: imported whole fragrant rice; LWF: local whole fragrant rice; LBFf: local broken fragrant‐free rice; IBFf: imported broken fragrant‐free rice; LsiBFf: local semi‐industrial broken fragrant‐free rice)

The intensity of some specific sensory descriptors such as color, grain size, and degree of cooking, cited as important for rice by women during FGDs, was subsequently evaluated by consumers to check whether the five different rice samples were just about right (JAR). Regarding the color descriptor (Figure 1a), respectively, 97% and 90% of consumers interviewed found the whole fragrant rice (local and imported) just about right (JAR), followed by the local broken fragrant‐free rice (LBFf) assessed JAR by 86% of the consumers. The whiteness of the grain is known to be highly appreciated by consumers (Mestres et al., 2019) and can be affected by the inherent color of a given variety (Juliano, 2003). The color of the imported broken fragrant‐free rice (IBFf) and the local semi‐industrial one (LsiBFf) was found by, respectively, 55% and 48% of the respondents, to be too dark. Their lower whiteness can mainly be explained by the processing conditions, and the presence of some impurities. Indeed, the local broken rice was milled semi‐industrially by small‐scale rice millers, with the sorting, dehusking, and milling operations probably conducted in lower quality conditions. Consequently, the color of the rice resulting from a full industrial process was more appreciated than that of the semi‐industrial rice. In addition, as rice grains age, the color changes and becomes yellowish due to oxidation of phenols, leading to the formation of melanoidins (Jungtheerapanich et al., 2017; Park et al., 2012; Sirisoontaralak & Noomhorm, 2007). This could explain the darker color of the imported industrial broken rice (IBFf) which may have been stored for a long time before being exported and consumed. These results confirm those we obtained with the hedonic overall liking test. Indeed, the rice with the most appreciated color (JAR) happened to be the most liked one, with the highest overall liking score of 7.7 (Group A). Color seems to have a significant impact on the choice of rice samples by women consumers. Figure 1b shows the results of the JAR test on the degree of cooking. The same two whole fragrant rice samples (IWF and LWF) were scored JAR with regard to the degree of cooking (well‐cooked) by, respectively, 87% and 84% of consumers, for the imported and the local one. The other three samples were considered too pasty by an average of 30% of consumers. These results show that, for some consumers, broken rice was perceived as being overcooked compared with whole rice. The degree of cooking could depend on the grain size, since the quantity of water added was the same for each sample. Vidal et al. (2007) showed that the cooking time increases significantly with the width of the kernel and that it takes longer to cook whole rice than broken rice. Our results also show that the degree of cooking seems to have an impact on the overall liking since the broken rice samples were scored lower in the hedonic tests (groups B, BC, and C, from 5.9 to 6.7). The degree of cooking (well‐cooked) seems to have a positive impact on the choice of rice. Figure 1c shows the results of the JAR test on grain size. A majority of consumers (73 to 88%) found the grain size of all the five rice samples to be JAR, in particular for LBFf and LWF, the local industrial broken and whole rice (88 and 86% of consumers, respectively). However, respectively, 27% and 13% of consumers found the grain size of the imported and local whole rice sample (IWF and LWF) too large. On the other hand, respectively, 27% and 23% of consumers thought that the grain size of the imported broken rice sample (IBFf) was too small and the local semi‐industrial broken rice (LsiBFf) was too big, probably because it is composed of a mixture of whole grains and broken grains at 50% (heterogeneous grain size). The women living in the Saint‐Louis region of Senegal evaluated broken rice and whole rice in the same way since they use both for making different popular meals (*ceebu jen* and *yassa,* respectively). However, the homogeneity of the grain size of the rice samples seemed to them to be an important sensory characteristic, and it was one of the reasons they scored their overall liking of the local semi‐industrial broken rice (LsiBFf) lower.

The descriptors color (white), degree of cooking (well‐cooked), and grain size (homogeneous size) had a significant impact on the choice of rice samples by the women consumers, which was confirmed by the overall liking scores. However, the differentiation between rice samples regarding the degree of cooking and the grain size was less clear than it was for the color descriptor.

### 4.5 Mapping of the CATA sensory descriptors with hedonic overall liking of the rice samples

**FIGURE 2:**
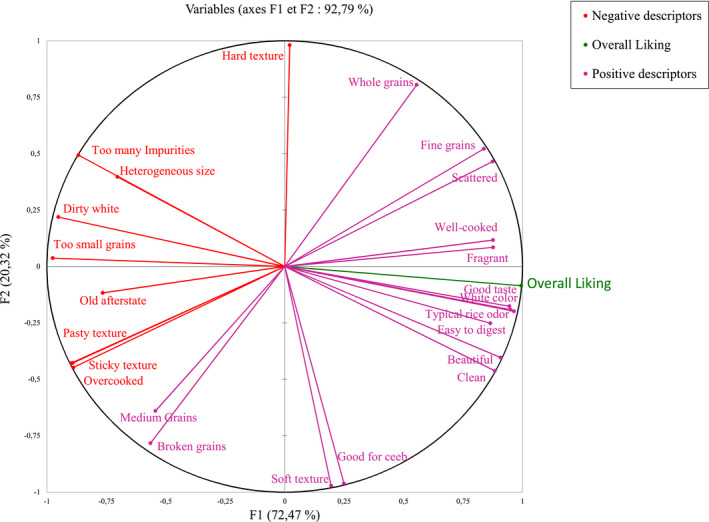
Multifactorial Analysis on sensory descriptors and overall liking of rice samples

Table 5 lists the frequency of citations of the CATA descriptors used by the 120 women consumers to describe the five rice samples during the interviews in three different districts in the city of Saint‐Louis. The Q Cochran's test revealed significant differences in the frequency of citations of each descriptor used by consumers to describe each rice sample, with p‐value < 0.01, below the significance level (*p* < .05), suggesting that consumers perceived differences between the rice samples in terms of their sensory characteristics. Next, a multifactorial analysis (MFA) was conducted on these data (Figure 2). The projection on this plan represents 92.8% of the variability, with axis 1 explaining 72.5% of the variability opposing the most liked and least liked descriptors. Some quality descriptors such as fine grains, scattered, well‐cooked, fragrant, typical rice odor, good taste, white color, easy to digest, beautiful, and clean were strongly and positively correlated with the high overall liking scores. On the other hand, descriptors such as too many impurities, heterogeneous size, dirty white, too small grains, old aftertaste, sticky texture, pasty texture, and overcooked were positively correlated with each other, and negatively correlated with most positive descriptors and high overall liking scores. These results confirm what was said by the women interviewed during the two focus group discussions. The women did not like overcooked sticky rice, with many impurities, these being negative descriptors related to lower rice quality. Conversely, they emphasized that the criteria for choosing a high‐quality rice are linked to positive descriptors such as ease of cooking, white color, cleanliness (no impurities), uniformity (grain size), and homogeneous shape of rice grains. These results also confirm those obtained with the JAR test on the three descriptors, color, degree of cooking, and grain size of rice. The JAR test results showed that the descriptors white color, well‐cooked, and homogeneous grain size are the criteria that are positively linked with higher overall liking of the rice samples. Similar studies have also been carried out in other African countries, and the results obtained were similar to ours. In Northern Ghana, the criteria that guide consumer choice are taste, aroma, cooking time, absence of impurities, and affordable prices (Anang et al., 2011). The Nigerien consumers prefer locally milled rice over imported rice because it is cheaper and tastes better (Abdullahi et al., 2019). The CATA test results obtained in this study complement previous studies (Demont et al., 2013; Fall et al., 2007). Axis 2, which explains 20.3% of the variability, contrasts the descriptors such as good for *ceeb*, whole grains, medium grains, broken grains, and hard and soft texture, which distinguished rice samples, but were not linked to their overall liking.

**TABLE 5:** Frequency of citations by consumers (*n* = 120) of each descriptor for each product

Descriptors	LsiBFf	LWF	LBFf	IBFf	IWF
Broken grains ***	6	2	89	105	2
Whole grains ***	101	103	8	2	109
Pasty texture***	54	30	64	72	13
Heterogeneous size***	61	29	36	28	12
Medium grains***	49	39	88	56	23
Easy to digest***	89	109	96	95	102
Hard texture***	27	12	7	7	20
Good taste***	86	113	103	84	109
Fine grains***	38	69	9	10	93
Too many impurities***	69	3	13	38	1
Too small grains***	27	2	13	32	1
Soft texture**	98	111	114	112	106
Fragrant***	32	41	41	30	58
White color***	39	115	91	41	115
Overcooked***	17	10	20	26	4
Sticky texture***	69	55	75	80	41
Well‐cooked**	92	107	92	97	109
Old aftertaste***	18	3	8	40	7
Scattered***	61	79	50	47	93
Typical rice odor ***	93	113	107	92	112
Clean***	62	119	111	83	119
Good for ceeb***	91	105	111	104	100
Beautiful***	74	117	110	86	117
Dirty white ***	80	5	27	79	8

Note

In blue: Positive descriptors In red: Negative descriptors

** *p* < .01, ***p < .001 according to Q Cochran's test

### 4.6 Sensory characteristics of the cooked rice samples

**FIGURE 3:**
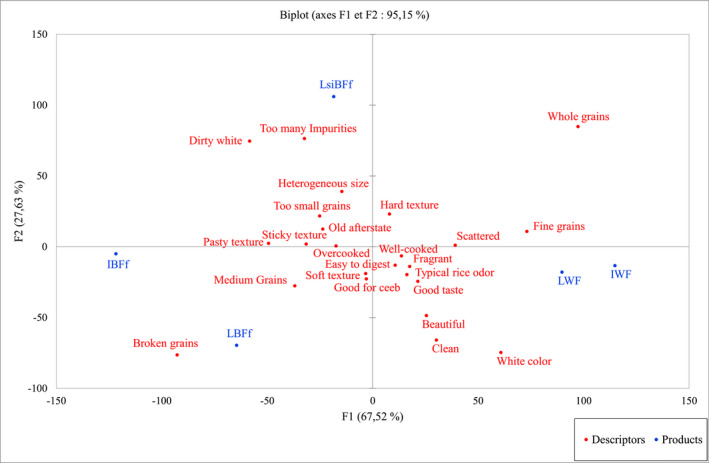
Principal component analysis of sensory descriptors of the rice samples (IWF: imported whole fragrant rice; LWF: local whole fragrant rice; LBFf: local broken fragrant‐free rice; IBFf: imported broken fragrant‐free rice; LsiBFf: local semi‐industrial broken fragrant‐free rice)

A PCA was performed on the frequency of citations of the CATA descriptors (Table 5) chosen by consumers to describe the five rice samples. The projection of the CATA data with the rice samples (Figure 3) accounted for 95.2% of the total variation. Axis 1 explains 67.5% of variability and allowed the rice samples to be divided into four groups. Group 1 consists of the local and imported industrial whole fragrant rice samples (LWF and IWF), which were described by positive CATA descriptors such as whole grain, fine grain, scattered, well‐cooked, fragrant, typical rice odor, good taste, and easy to digest. Group 2 is composed of local industrial broken rice (LBFf) that was described by positive descriptors such as good for *ceeb*, soft texture, clean, and beautiful. Group 3, composed of the imported industrial broken rice (IBFf), was characterized by descriptors such as broken grains and medium grains, dirty white, too small grains, old aftertaste, sticky texture, pasty texture, overcooked, and broken grains. Finally, group 4 is composed of the local semi‐industrial broken rice (LsiBFf), which is described by negative descriptors such as too many impurities, heterogeneous size, and hard texture. These results confirmed the classification of rice samples by Tukey's test on hedonic overall liking. The same rice samples associated with the positive descriptors were described as JAR by more consumers for their color, degree of cooking, and grain size. On the other hand, the rice samples associated with the negative descriptors were those that were found to be too pasty, not homogeneous in size and darker in color by consumers in the JAR test. Among these samples, the local semi‐industrial broken rice (LsiBFf) and the imported industrial broken rice (IBFf) were more associated with negative descriptors linked to poor technological processing and storage conditions, namely the presence of impurities, dirty white, heterogeneous size, and old aftertaste.

Our results confirmed that industrially and properly processed local rice can compete with imported rice in Saint‐Louis (Senegal); this was observed without specific labeling of local rice which improved their acceptability (Demont et al., 2013). Local rice acceptability is linked to good processing conditions, and criteria such as grain size homogeneity, whiteness (as opposed to dirty white), and cleanliness of the local rice must meet international standards. Indeed, efforts have already been made in West Africa in recent years to improve rice postharvest technology and to accompany changing consumption patterns (Fiamohe et al., 2018; Nwachukwu & Achike, 2020). Considerable investments have been made in semi‐industrial and industrial milling technologies; for example, in 2019, 57 rice mills with semi‐industrial or industrial technologies with an aggregate capacity of 315 tons per hour were operating in West Africa (Soullier et al., 2020). In the case of Nigeria, different quality‐enhancing postharvest technologies have been developed and disseminated to rice farmers. These technologies include using appropriate harvesting techniques such as threshing, winnowing, drying, improved parboiling, milling, storage, and even polishing of local rice to reduce wastage and improve the quality of the rice (Adisa et al., 2020). In Benin, methods for threshing and cleaning rice have been developed and evaluated with producers. These methods were effective in preserving the quality of the rice grains by increasing the efficiency of threshing and reducing the rate of breakage, which is a key factor influencing rice quality (Olaye et al., 2017). On the other hand, if processing is not done properly, dehusking and sorting, which are key steps for the removal of impurities, have to be done by hand prior to cooking. This is a disadvantage because in Africa, particularly in Senegal, women are no longer willing to waste time on those long intermediate steps (Demont et al., 2013).

As already pointed out by Demont et al. (2013) using experimental auctions, our results showed that the second type of characteristics that influence local rice acceptability is their intrinsic quality, more precisely: good taste, typical rice aroma, and fragrance, as opposed to old aftertaste. This study pointed to consumer preference for fragrant local or imported rice. Previous studies have already reported that the preference for fragrant rice was based on factors such as ethnicity, household size, and awareness of fragrance (Diagne et al., 2017). Fragrant rice varieties such as Jasmine and Basmati are popular not only in Senegal, but throughout the African continent (Bhattacharjee et al., 2002). In Benin, high‐quality white and fragrant rice is preferred in urban areas (Rutsaert et al., 2013). In this context, AfricaRice has developed three fragrant rice varieties Sahel 177, Sahel 328, and Sahel 329 since 2009 (Diagne et al., 2017) and more research is needed on breeding new varieties of fragrant rice as it is increasingly consumed in urban areas in Africa; for example, in Dakar (Senegal), three quarters of consumers are willing to upgrade from nonfragrant to fragrant rice, whether imported or not (Diagne et al., 2017).

Finally, this study also shows that consumers in Saint‐Louis prefer whole rice to broken rice. However, this depends on the dish. Indeed, in the multifactorial analysis (MFA) the second axis opposed the variables “whole grain” and “broken grains” and it also showed that “broken grains” was correlated with “good for *ceeb*.” These results confirm that in Senegal, broken rice is mainly used to prepare “*ceeb,*” which is a “pilaf” way of cooking rice in a sauce or broth, in contrast to the whole grain cooked in water.

## 5 CONCLUSION

This original study conducted with a large number of women consumers in Saint‐Louis produced exclusive information on the quality characteristics of the local rice compared to imported rice. The main conclusion of the study is that local rice can compete with imported rice if processing is improved. This will be the first step in improving rice quality to match consumer expectations, mainly in the case of semi‐industrial samples, thereby enhancing the branding of the local rice variety, the so‐called “*riz de la vallée*.” This study in Saint‐Louis can now be extended to other regions of Senegal to get an overview of consumer demand across the country as this may differ considerably from north to south. The relationships between consumer acceptance and physical–chemical characteristics of the rice grain will be published in a following paper.

## ETHICAL STATEMENT

This study is conform to the European Medicines Agency Guidelines for human subjects, and the consumer tests were approved by the Ethics Committee of the Cheikh Anta Diop University of Dakar. The authors have no conflicts of interest to declare.

## Data Availability

Data available on request from the authors.
